# Prognostic Value of HIF-1α-Induced Genes in Sepsis/Septic Shock

**DOI:** 10.3390/medsci11020041

**Published:** 2023-06-12

**Authors:** Nikolaos S. Lotsios, Chrysi Keskinidou, Edison Jahaj, Zafeiria Mastora, Ioanna Dimopoulou, Stylianos E. Orfanos, Niki Vassilaki, Alice G. Vassiliou, Anastasia Kotanidou

**Affiliations:** 1 1st Department of Critical Care Medicine & Pulmonary Services, School of Medicine, National and Kapodistrian University of Athens, Evangelismos Hospital, 10676 Athens, Greece; n.lotsios96@gmail.com (N.S.L.); chrysakes29@gmail.com (C.K.); edison.jahaj@gmail.com (E.J.); zafimast@yahoo.gr (Z.M.); idimo@otenet.gr (I.D.); stylianosorfanosuoa@gmail.com (S.E.O.); 2 Laboratory of Molecular Virology, Hellenic Pasteur Institute, 11521 Athens, Greece; nikiv@pasteur.gr

**Keywords:** HIF1A, sepsis, septic shock, ICU, HMOX1

## Abstract

Hypoxia is characterized as one of the main consequences of sepsis, which is recognized as the leading cause of death in intensive care unit (ICU) patients. In this study, we aimed to examine whether the expression levels of genes regulated under hypoxia could be utilized as novel biomarkers for sepsis prognosis in ICU patients. Whole blood expression levels of hypoxia-inducible factor-1α (*HIF1A*), interferon-stimulated gene 15 (*ISG15*), hexokinase 2 (*HK2*), lactate dehydrogenase (*LDHA*), heme oxygenase-1 (*HMOX1*), erythropoietin (*EPO*), and the vascular endothelial growth factor A (*VEGFA*) were measured on ICU admission in 46 critically ill, initially non-septic patients. The patients were subsequently divided into two groups, based on the development of sepsis and septic shock (n = 25) or lack thereof (n = 21). *HMOX1* mRNA expression was increased in patients who developed sepsis/septic shock compared to the non-septic group (*p* < 0.0001). The ROC curve, multivariate logistic regression, and Kaplan–Meier analysis demonstrated that *HMOX1* expression could be utilized for sepsis and septic shock development probability. Overall, our results indicate that *HMOX1* mRNA levels have the potential to be a valuable predictive factor for the prognosis of sepsis and septic shock in ICU patients.

## 1. Introduction

Sepsis is a complex disorder, defined as a life-threatening organ dysfunction attributed to dysregulated host response to infection [1]. In recent years, sepsis has become the main cause of mortality in intensive care units (ICUs) [2]. Septic shock is a subset of sepsis with a greater mortality risk, attributed to profound circulatory, cellular, and metabolic abnormalities [1]. Mitochondrial dysfunction and hypoxia have been linked to cell injury and sepsis-induced organ dysfunction, although to this day the exact molecular mechanisms in play have not been fully elucidated [3]. The discovery of novel markers with prognostic and/or diagnostic significance is crucial so that in combination with modern treatments, the outcome of septic patients can be improved [4]. 

In cases where oxygen levels are reduced, the hypoxia-inducible factors (HIFs) are responsible for regulating the cellular response [5]. HIFs are heterodimeric transcription factors, which consist of two subunits, an oxygen-sensitive (α-subunit) and a constitutively expressed subunit (β-subunit) [6]. HIF regulation under hypoxia is achieved in part through the negative feedback loop between itself and the interferon-stimulated gene 15 (ISG15) protein, a member of the ubiquitin family [7]. ISG15 is a secreted protein with a molecular weight of 15 kDa, strongly induced by type I interferons, with a main role in protein ISGylation [8]. HIF stabilization due to hypoxia leads to the increased expression of ISG15, which in turn neutralizes the formation of the HIF-1α heterodimer. In sepsis, HIF-1α has been described as a key component in numerous phases of inflammatory responses [9]. In addition to inflammation, HIF-1α exerts a significant effect on cell metabolism, regulating the expression of genes related to glycolytic metabolism. During sepsis, HIF-1α, in combination with other driving factors, is responsible for the metabolic switch of immune cells from oxidative phosphorylation to glycolysis, an event known as the Warburg effect, enabling cell proliferation and the cytokine storm described in sepsis [10]. Hexokinase 2 (*HK2*) and lactate dehydrogenase (*LDHA*) are among the glycolytic genes induced by HIF-1α [11,12,13]. HK2 is the first rate-limiting enzyme of glycolysis, catalyzing the phosphorylation of glucose to glucose-6-phosphate (G-6P), while LDHA is responsible for the reversible enzymatic conversion of pyruvate to lactate [14,15]. 

Except for enzymes contributing to metabolic pathways, under hypoxia, HIFs regulate several genes with central roles in sepsis, including heme oxygenase-1 (*HMOX1*), erythropoietin (*EPO*), and the vascular endothelial growth factor (*VEGFA*) [16,17,18,19]. Heme oxygenase (HO-1) scavenges heme that is released into the circulatory system during sepsis-induced red blood cell lysis and subsequent oxidation of hemoglobin [20]. By degrading heme, HO-1 exerts a potent cytoprotective role in the lungs [21]. Recent studies have demonstrated that HO-1 alleviates sepsis-induced acute lung injury by inhibiting Golgi stress, as well as endoplasmic reticulum stress [22,23]. 

Furthermore, EPO regulates bone marrow erythropoiesis via interaction with the surface receptor EPO-R, and its potential utility as a therapeutic treatment in sepsis has been rigorously studied, presenting a strong anti-apoptotic, cytoprotective, and anti-inflammatory effect in several animal models [24,25,26,27,28,29,30]. Lastly, VEGFA retains central roles in cell migration, as well as in vessel formation and permeability [31]. Recent evidence supports that the excessive release of VEGFA from pulmonary vascular endothelial cells could be associated with the development of non-cardiogenic pulmonary edema in sepsis-associated lung injury [32]. 

The aim of this study was to investigate the gene expression profile of *HIF1A*, *ISG15*, *HK2*, *LDHA*, *HMOX1*, *EPO,* and *VEGFA* under the setting of sepsis and septic shock, and furthermore to evaluate whether any of these molecules has potential value as a prognostic factor. To this end, we examined the mRNA expression levels in whole blood samples of patients admitted to the ICU who developed sepsis and septic shock or not. 

## 2. Materials and Methods

The “Evangelismos” Hospital Research Ethics Committee (80/01-02-2010) approved this observational study. All study procedures were performed after obtaining informed written consent from the patients’ next of kin, and were in accordance with the Helsinki Declaration. 

### 2.1. Characteristics of the Study Population

**Figure 1:**
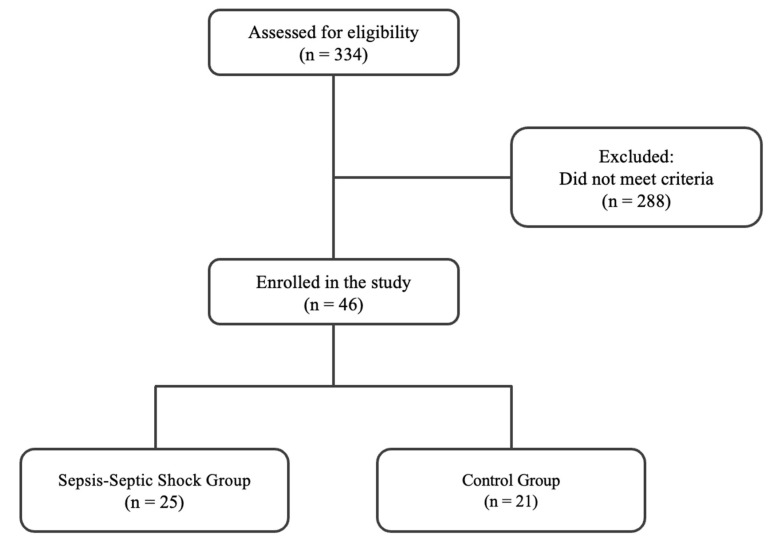
Flow chart of patient enrollment.

The flow chart of the enrolment is depicted in Figure 1. The screening took place from 20 September 2010 to 15 February 2016. The exclusion criteria were as follows: sepsis on ICU admission or sepsis development within the first 48 h post-ICU admission, age < 18, positive pregnancy test, presence of malignant neoplasm, brain death, contagious diseases (human immunodeficiency virus and hepatitis), ICU length of stay < 3 days, no need for intubation, transfer from another ICU, and oral intake of corticosteroids at an equivalent dosage of ≥1 mg/kg prednisone/day for a period greater than one month. In total, 334 ICU patients were initially screened for eligibility over the study period. Enrolled patients were assigned to two groups based on the development of sepsis and septic shock during their ICU stay, according to the Sepsis-3 guidelines [1]. Sepsis is defined as life-threatening organ dysfunction caused by a dysregulated host response to infection (increase in the sepsis-related sequential organ failure assessment (SOFA) score of 2 points or more). Septic shock is a subset of sepsis in which patients are identified by a vasopressor requirement to maintain a mean arterial pressure of 65 mm Hg or greater and serum lactate levels >2 mmol/L in the absence of hypovolemia. Those who did not develop sepsis during their ICU stay (*non-septic patients*) constituted the control group, while patients who developed it were assigned to the sepsis and septic shock group (*septic patients*).

### 2.2. Total RNA Extraction

Venous blood samples were obtained at various time points (Tempus RNA tubes, Thermo Fisher Scientific, Waltham, MA, USA). The first blood sample was drawn on ICU admission (within 24 h). For patients who subsequently developed sepsis and septic shock, 2 more blood samples were obtained within 6 h of their development, respectively. For non-septic patients, a second blood sample was drawn at ICU discharge. The corresponding kit was utilized to isolate total RNA from the peripheral blood, per the manufacturer’s instructions. The concentration and quality of isolated total RNA were measured with a spectrophotometer at 260/280 nm. 

### 2.3. Reverse Transcription and Quantitative Real-Time PCR

cDNA was synthesized from the isolated total RNA (Nippon Genetics, Duren, Germany), and the quantitative real-time PCR (qPCR) method (Kapa SYBR^®^ Green PCR Master Mix, Sigma-Aldrich, St Louis, MO, USA) was used to measure *HIF1A*, *HMOX1*, *EPO*, *ISG15*, *LDHA*, *HK2,* and *VEGFA* mRNA expression using the specific primer sets listed in Table 1.

Quantitative real-time PCR was performed in 96-well plates (CFX Connect thermocycler, Biorad, Hercules, CA, USA). The comparative CT method 2^−ΔΔCT^ [33] was applied using *GAPDH* mRNA expression levels for normalization of the target gene expression levels. ICU admission values within each group were used as the control. 

### 2.4. Statistical Analysis

Data are presented either as individual values, mean ± SD for normally distributed data, or median with interquartile range (IQR) for skewed data. Comparisons between the two groups were performed using Student’s *t*-test, the non-parametric Mann–Whitney test, the paired non-parametric Wilcoxon test, or the chi-square test, as appropriate. Comparisons between three groups were performed using the paired non-parametric Friedman test. Spearman’s coefficient was used to test correlations. A receiver operating characteristic (ROC) curve was generated using the development of sepsis/septic shock in the ICU or its absence as the classification variable, and *HMOX1* DCt values on ICU admission as the prognostic variable. In qPCR, Ct values represent the number of amplification cycles required to reach the signal threshold. DCt is defined as the difference between the Ct value of the target gene (*HMOX1*) and the control gene (*GAPDH*). The optimal cut-off value for risk for sepsis/septic shock onset prediction was calculated as the point with the greatest combined sensitivity and specificity (Youden index). Univariate and multivariate logistic regression analyses were performed to identify potential risk factors for sepsis/septic shock development in the ICU. The whole patient cohort was dichotomized above and below the cut-off value determined from the ROC curve, and the Kaplan–Meier method was subsequently used for sepsis/septic shock probability estimation, using the log-rank test for comparison. All *p*-values are two-sided; significance was set at *p* < 0.05. 

## 3. Results

### 3.1. Characteristics of the Study Population

A total of 46 (32 male and 14 female) critically ill, mechanically ventilated, initially non-septic patients were enrolled in the study. These patients suffered from surgical, medical, and trauma-related pathologies. A total of 25 patients (19 male and 6 female) subsequently developed sepsis (on day 5 (IQR: 3–8)) and septic shock (on day 12 (IQR: 8–17)) during their ICU stay (*septic patients),* while the remaining 21 patients (13 male and 8 female) did not develop sepsis during their ICU stay (*non-septic patients)* and were used as a control group. All 25 patients who developed sepsis/septic shock had positive biological fluid cultures. In 23 of our septic patients (92%), sepsis originated from a lung infection. The infections were mainly due to Gram-negative bacteria (88%, mostly *Acinetobacter baumannii* and *Klebsiella pneumoniae*). Clinical data and blood samples were acquired from all the patients enrolled. The characteristics of the two patient groups upon admission are presented in Table 2. In septic patients, the SOFA score was higher. Additionally, the same group presented an extended duration of mechanical ventilation and length of stay in the ICU in comparison to non-septic patients.

### 3.2. mRNA Expression of Hypoxia-Regulated Genes in ICU Patients

**Figure 2:**
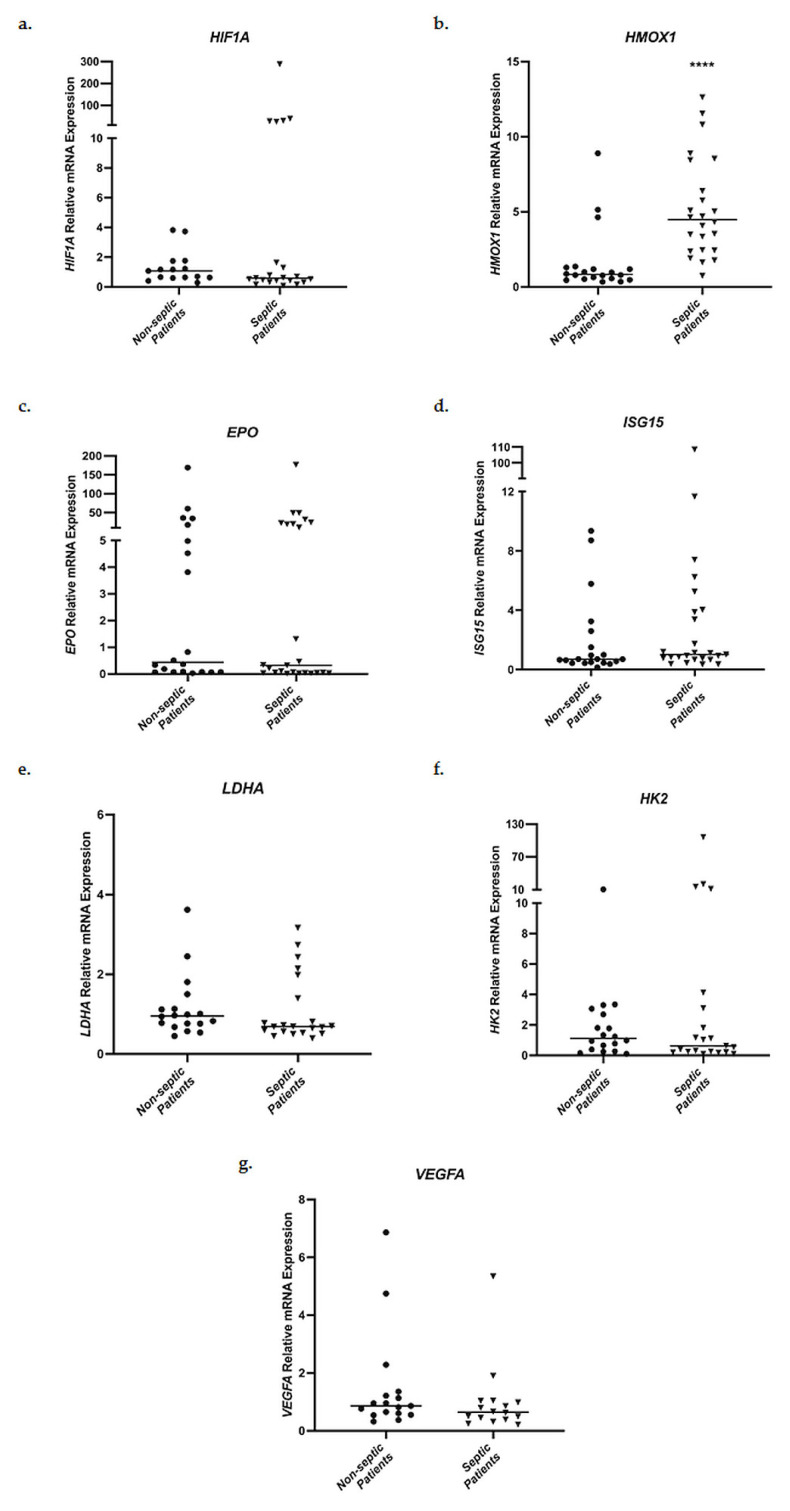
Distribution of *HIF1A* (**a**), *HMOX1* (**b**), *EPO* (**c**), *ISG15* (**d**), *LDHA* (**e**), *HK2* (**f**), and *VEGFA* (**g**) relative mRNA expression in whole blood samples on ICU admission of non-septic patients and septic patients. Data are presented as scatter plots. Line in the middle: median. **** *p* < 0.0001 vs. non-septic patients by the Mann–Whitney test.

Relative messenger RNA expression levels of *HIF1A*, *HMOX1*, *EPO*, *ISG15*, *LDHA*, *HK2,* and *VEGFA* in whole blood samples were evaluated in the two groups of patients admitted to the ICU, septic patients and non-septic patients. Initially, ICU admission mRNA levels were compared between the two groups (Figure 2). No statistically significant differences in mRNA expression were observed for *HIF1A*, *EPO*, *ISG15*, *LDHA*, *HK2,* and *VEGFA*. However, septic patients presented significantly elevated *HMOX1* mRNA levels on ICU admission, in comparison to non-septic patients (Figure 2b). Specifically, septic patients showed a 4.48-fold increase (interquartile range (IQR), 2.44–7.93) in *HMOX1* mRNA expression in comparison to 0.83-fold (0.54–1.26, *p* < 0.0001) in the non-septic patient control group.

Additionally, the mRNA expression levels of the above-mentioned genes were measured on ICU admission, sepsis, and septic shock for the septic group, and on admission and discharge for the non-septic group (Supplementary Materials, Figure S1). *HIF1A* mRNA levels exhibited a 0.69-fold (0.43–0.96; *p* < 0.05) decrease at septic shock compared to values on admission (Figure S1a). On the contrary, *HK2* mRNA levels presented a 1.56-fold (0.92–2.63; *p* < 0.05) increase at septic shock (Figure S1e). The non-septic patients exhibited decreased *HK2* and *LDHA* mRNA expression at discharge. Specifically, *LDHA* mRNA expression (Figure S1d) showed a 0.82-fold (0.60–0.89; *p* < 0.001) decrease, while for *HK2* (Figure S1e) we calculated a 0.77-fold decrease (0.51–1.08; *p* < 0.05). 

### 3.3. HMOX1 mRNA Expression as a Prognostic Factor for Sepsis and Septic Shock

**Figure 3:**
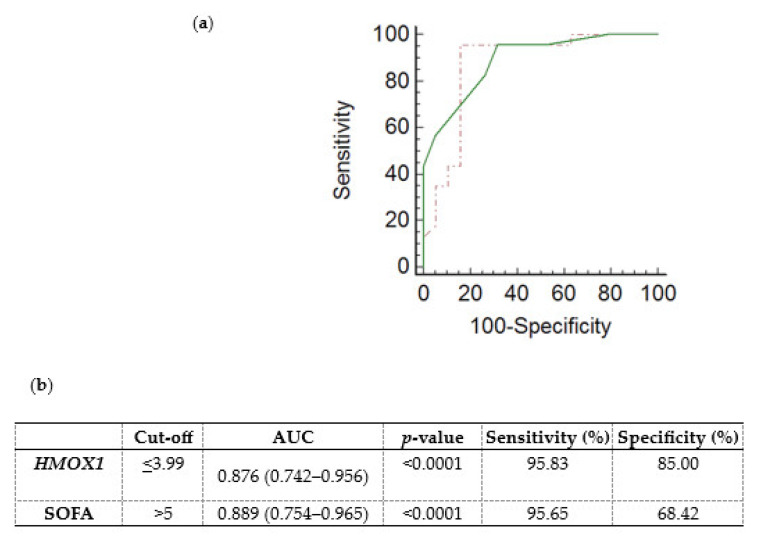
Whole blood *HMOX1* mRNA expression levels and probability of sepsis/septic shock development in critically ill, initially non-septic patients. (**a**) Receiver operating characteristic (ROC) curves were generated for *HMOX1* DCt values (dashed red line) and the SOFA score (solid green line) for the probability of sepsis/septic shock development in initially non-septic patients admitted to the ICU. (**b**) Table listing the cut-off values (Youden index), the area under the curve (AUC), and the sensitivity and specificity for *HMOX1* and SOFA score of the respective ROC curves.

A receiver operating characteristic (ROC) curve was generated, using the ICU admission *HMOX1* DCt values, to determine whether *HMOX1* levels could predict the subsequent development of sepsis and septic shock in critically ill, initially non-septic patients. Non-septic patients exhibited higher DCt values (4.97; (4.36–5.60)), and thus lower mRNA expression, in comparison to septic patients (2.54; (1.72–3.42), *p* < 0.0001). The ROC curve of sepsis/septic shock development probability presented an area under the curve (AUC) equal to 0.876 (0.742–0.956, Figure 3a,b; *p* < 0.0001). The cut-off was determined by the Youden index at ≤3.99. The generated ROC curve was compared with the ROC curve of the SOFA score (Figure 3a; *p* = 0.83).

Furthermore, a logistic regression analysis was performed to investigate the possible association between ICU admission *HMOX1* expression levels and the risk of sepsis/septic shock development (Table 3). The univariate model showed that lower *HMOX1* DCt values, corresponding to higher mRNA expression, correlated with an increased risk of sepsis/septic shock development (odds ratio (O.R.) = 0.259, 95% C.I. = 0.125–0.534, and *p* < 0.0001). Multivariate analysis was performed to correct for potential confounding factors, including SOFA score, age (continuous variables), and sex (categorical). According to our results, lower *HMOX1* DCt values may serve as a stand-alone marker for sepsis/septic shock development (adjusted O.R. = 0.258, 95% C.I. = 0.097–0.686, and *p* = 0.007).

**Figure 4:**
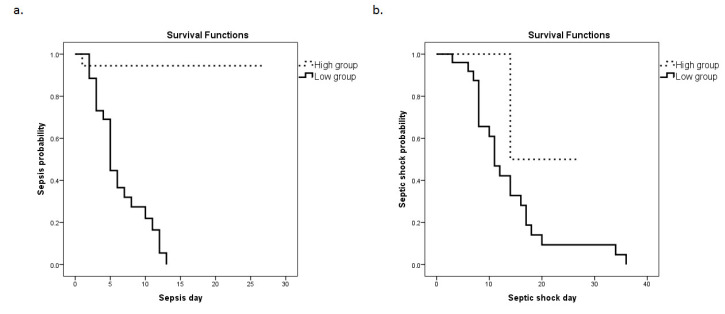
*HMOX1* expression levels and sepsis or septic shock development probability. The Kaplan–Meier method was used for sepsis (**a**) and septic shock (**b**) probability estimation, and the log-rank test for two-group comparison. The patient cohort was dichotomized above and below the cut-off value (3.99), as determined by the ROC curve. Dashed lines: ≥3.99 (high group); solid lines: <3.99 (low group). (**a**) The respective median time to sepsis development was 6 (95% C.I. = 5–8) days for the low group, and 25 (23–28) days for the high group (*p*-value < 0.0001). (**b**) The respective median times to septic shock development were 13 (10–19) days for the low group and 21 (11–30) days for the high group (*p*-value = 0.04).

Subsequently, a Kaplan–Meier analysis was performed (Figure 4a,b). The ICU cohort was dichotomized above (high group) and below (low group) the cut-off value determined from the ROC curve using the Youden index (3.99). The probability for the development of sepsis and septic shock was significantly elevated in the low groups (corresponding to higher mRNA expression). Specifically, the median time for sepsis development was 6 (5–8) days following admission to the ICU for the *HMOX1* low group in comparison to 25 (23–28) days for the *HMOX1* high group (*p* < 0.0001). For septic shock development, the median time for the *HMOX1* low group was 13 (10–19) days, whereas for the *HMOX1* high group, it was 21 (11–30) days (*p* = 0.04).

### 3.4. Correlations of HIF-Regulated Gene Expression on ICU Admission

**Figure 5:**
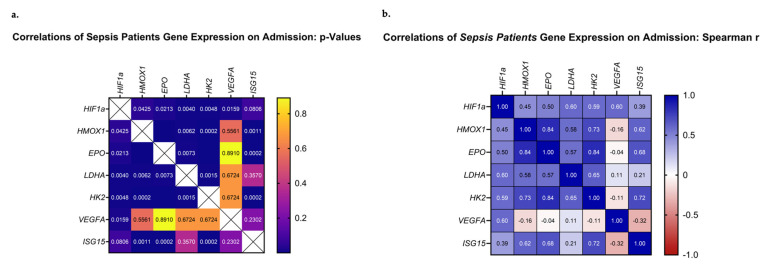
Correlations of *HIF1A*, *HMOX1*, *EPO*, *LDHA, ISG15*, *HK2,* and *VEGFA* relative mRNA expression on ICU admission. (**a**) The *p*-values and (**b**) Spearman r values of each pair are presented as heat maps.

The next step in our analysis was to evaluate whether the above-mentioned genes presented any correlations between their mRNA expression patterns. Our analysis focused on the mRNA expression levels of the septic patient group. Upon admission, all studied genes, except for *VEGFA*, correlated significantly with each other and demonstrated positive Spearman coefficient correlation values (Figure 5a,b). *VEGFA* mRNA levels correlated only with *HIF1A* (*p* = 0.002, r_s_ = 0.60). Most importantly, the highest Spearman values were calculated for pairs involving *EPO*, *HMOX1, HK2,* and *ISG15* (*EPO*–*HMOX1*, *p* < 0.0001, r_s_ = 0.84; *EPO*–*HK2*, *p* < 0.0001, r_s_ = 0.84; *HMOX1*–*HK2*, *p* = 0.0002, r_s_ = 0.73, *ISG15* –*HK2*, *p* = 0.0002, r_s_ = 0.72; *ISG15*–*EPO*, *p* = 0.0002, r_s_ = 0.69; and *ISG15*–*HMOX1*, *p* = 0.0011, r_s_ = 0.62). Additionally, pairs involving *HIF1A*, *LDHA,* and *HK2* presented high Spearman values (*HIF1A*–*LDHA*, *p* = 0.006, r_s_ = 0.60; *HIF1A*–*HK2*, *p* = 0.005, r_s_ = 0.59; and *LDHA*–*HK2*, *p* = 0.002, r_s_ = 0.65). Lastly, worth noting is the fact that *ISG15* mRNA expression demonstrated a positive correlation with the day of sepsis development (*ISG15*–day of sepsis, *p* = 0.035, r_s_ = 0.42), while it tended to correlate with the day of septic shock development (*ISG15*–day of septic shock, *p* = 0.059, r_s_ = 0.39). The same trend was observed for *EPO* mRNA expression (*EPO*–day of sepsis, *p* = 0.10, r_s_ = 0.34; *EPO*–day of shock, *p* = 0.08, r_s_ = 0.36). A larger cohort of patients could prove beneficial in elucidating the true relationship between the two.

## 4. Discussion

Hypoxia is established as one of the main consequences of sepsis. Thus, the present study aimed to analyze whether genes with central roles in sepsis, known to be regulated by HIFs, exhibit a differential expression pattern in patients admitted to the ICU. Our study focused on analyzing gene expression patterns during admission, sepsis, and septic shock, as well as investigating differences on admission among initially non-septic patients who eventually developed or not sepsis/septic shock in the ICU. Our findings suggest that *HMOX1* mRNA expression on ICU admission could be useful as a prognostic marker for sepsis and septic shock development. 

To this day, few studies have explored heme oxygenase expression in septic patients and its importance as a prognostic factor. In these studies, the serum or plasma levels of heme oxygenase (HO-1) protein were measured, with little to no data regarding *HMOX1* mRNA expression in sepsis patients. Xia et al. studied a cohort of septic and non-septic patients suffering from acute kidney injury (AKI) and demonstrated that the septic-AKI group presented elevated serum HO-1 protein levels in comparison to the non-septic-AKI group. They proposed that the extent of AKI progression in septic patients could be predicted by the increase in serum HO-1 levels [34]. Additionally, a study performed by Chen et al. demonstrated that in a cohort of COVID-19 patients, serum HO-1 protein levels were significantly elevated in those suffering from sepsis. When the septic patients were divided into survivors and non-survivors, the latter subgroup presented higher serum HO-1 levels [35]. Furthermore, another recent study showed that in a group of 70 patients diagnosed with sepsis, high HO-1 plasma levels measured on admission were significantly associated with disease severity and mortality [36].

In our cohort of ICU-admitted patients, we found that *HMOX1* mRNA expression on ICU admission was significantly elevated in the septic patient group. ROC curve analysis in combination with multivariate regression analysis indicated that *HMOX1* levels could be used as an independent prognostic factor for patients with a higher risk of developing sepsis and septic shock. Furthermore, the Kaplan–Meier analysis demonstrated that patients with higher *HMOX1* expression have a higher probability of developing sepsis and septic shock compared to those with a lower expression.

Many studies have indicated a potential prognostic role for several of the molecules investigated in this report. Tamion et al. presented results indicating that in a cohort of 44 septic patients, EPO serum levels on admission were able to predict the outcome of patients [37]. Our results depict a tendency for correlation of EPO mRNA expression with the day of sepsis and septic shock development. Thus, a possible argument arises in relation to the use of EPO as a therapeutic treatment strategy. It could be of interest to examine whether the effect of EPO administration is correlated with the patients’ endogenous levels of EPO expression. Furthermore, *ISG15* mRNA expression levels correlated positively with the day of sepsis development, while tending to correlate with the day of septic shock development. Additionally, in patients who developed sepsis, *EPO* and *ISG15* mRNA expression levels at the time of ICU admission were positively correlated. This could be potentially attributed to the involvement of ISG15 in erythropoiesis. Maragno et al. have shown that ISG15 expression is induced during the late stages of erythropoiesis, where EPO retains a crucial role [24,38]. In this context, and consistent with our findings, ISG15 induction is, at least partially, dependent upon EPO receptor signaling and mainly independent of IFN signaling.

The results from a recent meta-analysis have demonstrated that VEGFA levels can also accurately predict sepsis mortality, with non-survivors presenting higher values [39]. Additionally, regarding LDHA, a number of studies have demonstrated that serum lactate levels could be of value in predicting mortality [40,41]. Finally, even though HK2 has not been assigned any prognostic value in sepsis, the enzyme has been significantly associated with mortality in several types of cancer [42,43]. In contrast to these data on protein serum levels, expression of the above-mentioned molecules did not present significant differences between ICU-admitted patients who did or did not develop sepsis. It should be noted though that our study focused on gene expression levels and not the serum levels of their respective products. Despite not presenting an association with any outcome, we showed that all studied genes, except *VEGFA*, positively correlated on admission in patients who developed sepsis. These results suggest an interplay between these molecules in the setting of sepsis and septic shock and would be interesting to investigate in future larger studies. 

It is important to mention the limitations of our study. Initially, this was a single-center study with a limited number of patients. This limitation is derived from our choice of enrolling only patients who developed both sepsis and septic shock in the ICU. A cross-center study with a larger sample size might allow for the generalization of our results. Additionally, the septic patient group did not exhibit high mortality rates, and thus we could not examine the potential value of *HMOX1* mRNA expression as a predictive risk factor for ICU outcome. Furthermore, it should be noted that this was an observational study, and hence the mechanisms implicated in the expression of the studied genes were not investigated. *HMOX1* expression can be upregulated in response to various cellular stress stimuli, including inflammation, hypoxia–hyperoxia, hyperthermia, ischemia, or radiation.

## 5. Conclusions

As far as we are aware, this is the first report to assign to *HMOX1* expression a possible prognostic role for sepsis development. Our results indicate that ICU admission values of *HMOX1* gene expression could reveal patients at risk of developing sepsis and septic shock. Hence, the measurement of *HMOX1* gene expression on ICU admission may provide physicians with a useful tool for determining the likelihood of sepsis and septic shock occurrence, allowing for more targeted treatment.

## Figures and Tables

**Table 1:** Primer pairs used in quantitative real-time PCR experiments.

Gene		Sequence (5′-3′)	nt
*HIF1A*	F	5′-GGCGCGAACGACAAGAAAAAG-3′	21
	R	5′-CCTTATCAAGATGCGAACTCACA-3′	23
*HMOX-1*	F	5′-GGCCTAAACTTCAGAGGGGG-3′	20
	R	5′-AGACAGCTGCCACATTAGGG-3′	20
*EPO*	F	5′-GCCCCACCACGCCTCATCTGT-3′	21
	R	5′-CTTCCAGGCATAGAAATTAAC-3′	21
*ISG15*	F	5′-CCACCTGAAGCAAGCAAGTGA-3′	21
	R	5′-CGCAGGCGCAGATTCATGAA-3′	20
*LDHA*	F	5′-TTCACCCATTAAGCTGTCATGG-3′	22
	R	5′-GACACCAGCAACATTCATTCCA-3′	22
*HK2*	F	5′-GAGCCACCACTCACCCTACT-3′	20
	R	5′-ACCCAAAGCACACGGAAGTT-3′	20
*VEGFA*	F	5′-CTTGCCTTGCTGCTCTAC-3′	18
	R	5′-TGGCTTGAAGATGTACTCG-3′	19
*GAPDH*	F	5′-ATGGGGAAGGTGAAGGTCG-3′	19
	R	5′-TACATGAGGGCACGGAAGATG-3′	23

**Table 2:** Demographic information, clinical characteristics, and laboratory data of critically ill patients on ICU admission.

Characteristics	Septic Patients	Non-Septic Patients	*p*-Value
Number of patients, n	25	21	
Age (years), (mean ± SD)	51 ± 28	53 ± 18	0.76
Sex, n (%)			0.35
Male	19 (59.4%)	13 (40.6%)	
Female	6 (42.9%)	8 (57.1%)	
Diagnosis, n (%)			0.15
Surgical	3 (12.0%)	7 (33.3%)	
Medical	5 (20.0%)	5 (23.8%)	
Trauma	17 (68.0%)	9 (42.9%)	
APACHE II score (median, IQR)	15 (12–20)	14 (11–16)	0.16
SOFA score (median, IQR)	8 (7–10)	5 (3–7)	<0.0001 ****
PaO_2_/FiO_2_ (mmHg), (median, IQR)	360 (215–413)	381 (256–430)	0.32
Lactate (mmol/L) (median, IQR)	1.7 (1.0–2.9)	1.5 (0.8–1.7)	0.21
CRP (mg/dL), (median, IQR)	4.2 (1.6–8.9)	3 (1.4–8.5)	0.41
PCT (ng/mL), (median, IQR)	0.4 (0.1–1.7)	0.3 (0.1–0.8)	0.78
IL-6 (pg/mL), (median, IQR)	21.8 (8.6–64.4)	31.8 (8.4–75.6)	0.96
IL-8 (pg/mL), (median, IQR)	64.1 (23.4–102.3)	40.2 (20.0–137.3)	0.70
TNF-α (pg/mL), (median, IQR)	40.5 (29.3–73.6)	83.1 (50.6–173.1)	0.004 **
EPO-R (ng/mL) (median, IQR)	3.5 (1.1–10.3)	3.3 (1.5–6.9)	0.88
Day of Sepsis (median, IQR)	5 (3–8)	N/A	
Day of septic shock (median, IQR)	12 (8–17)	N/A	
Site of infection, n		N/A	
Pneumonia	23		
CNS	1		
Blood	1		
Type of infection, n		N/A	
Gram-negative bacteria	22		
Gram-positive bacteria	3		
Mechanical ventilation duration (median, IQR)	27 (19–38)	2 (1–4)	<0.0001 ****
Length of stay (median, IQR)	36 (24–47)	6 (3–10)	<0.0001 ****
ICU mortality, n (%)	8 (32%)	0 (0%)	0.005 **

Data are presented as individual numbers, n (%), mean ± SD, or median (IQR). Enrolled patients were assigned to one of two groups, septic patients and non-septic patients, based on the development of sepsis/septic shock, according to the Sepsis-3 guidelines. Comparisons between the two groups were performed using Student’s t-test, the Mann–Whitney test, or the chi-square test, as appropriate. Laboratory data were measured within 24 h of admission. APACHE, acute physiology and chronic health evaluation; CNS, central nervous system; CRP, C-reactive protein; EPO-R, erythropoietin receptor; ICU, intensive care unit; IL-6, interleukin 6; IL-8, interleukin 8; PCT, procalcitonin; SOFA, sequential organ failure assessment; TNF-α, tumor necrosis factor α. **, *p*-value < 0.01; ****, *p*-value < 0.0001.

**Table 3:** Results from the univariate and multivariate logistic regression analysis.

Variable	Univariate Model			Multivariate Model		
	O.R.	95% C.I.	*p*-Value	O.R.	95% C.I.	*p*-Value
SOFA score	2.635	1.456–4.770	0.001 *	2.919	1.257–6.775	0.013 *
*HMOX1*	0.259	0.125–0.534	<0.0001 *	0.258	0.097–0.686	0.007 *
Age	0.995	0.760–0.964	0.760	0.993	0.937–1.052	0.815
Sex	0.500	0.138–1.809	0.291	2.855	0.207–39.362	0.433

* *p*-value < 0.05. A univariate logistic regression model was fitted to examine the relationship of *HMOX1* mRNA expression levels with sepsis/septic shock development in the ICU. Multivariate regression analysis (backward method) revealed that *HMOX1* mRNA expression levels in the presence of a SOFA score could be assumed as independent indicators of sepsis/septic shock development (O.R. = 0.258 (95% C.I. = 0.097–0.686), *p* = 0.007 and O.R. = 2.919 (95% C.I. = 1.257–6.775), *p* = 0.013, respectively). C.I. confidence interval; O.R., odds ratio; SOFA, sequential organ failure assessment.

## Data Availability

Data are available upon reasonable request.

## Supplementary Materials

The following supporting information can be downloaded at https://www.mdpi.com/article/10.3390/medsci11020041/s1, Figure S1: Relative mRNA expression of *HIF1A* (a), *HMOX1* (b), *EPO* (c), *LDHA* (d), *HK2* (e), and *VEGFA* (f) in whole blood samples, on ICU admission, sepsis, and septic shock (septic patients), and on ICU admission and discharge (non-septic patients).
