# Supplementary material for: Prognostic Value of HIF-1α-Induced Genes in Sepsis/Septic Shock
Source: Med Sci (Basel). 2023 Jun 12;11(2):41. doi: 10.3390/medsci11020041 (PMC10300988; doi:10.3390/medsci11020041)
Supplement: Supplementary file 1 [file medsci-11-00041-s001.zip › medsci-2425554-supplementary.pdf]

medical sciences

MDPI

Supplementary Materials

Prognostic Value of HIF-1α-Induced Genes in Sepsis/Septic Shock

Nikolaos S. Lotsios, Chrysi Keskinidou, Edison Jahaj, Zafeiria Mastora, Ioanna Dimopoulou, Stylianos E. Orfanos, Niki Vassilaki, Alice G. Vassiliou and Anastasia Kotanidou

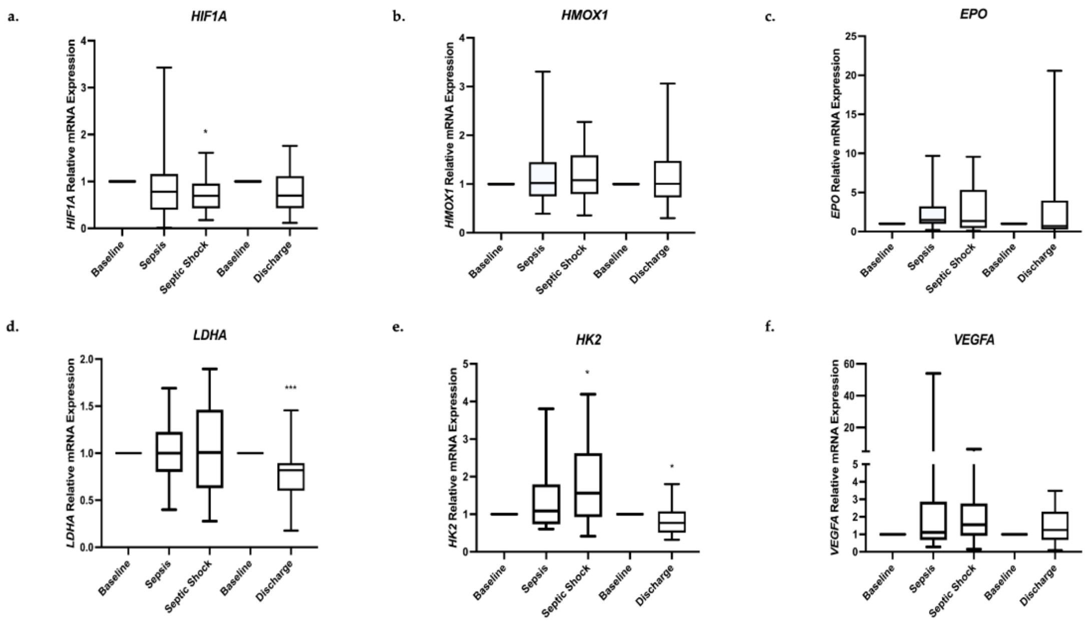

Figure S1: Relative mRNA expression of *HIF1A* (a), *HMOX1* (b), *EPO* (c), *LDHA* (d), *HK2* (e) and *VEGFA* (f) in whole blood samples, on ICU admission, sepsis and septic shock (septic patients), and on ICU admission and discharge (non-septic patients). The non-parametric Mann-Whitney test or the Kruskal-Wallis non-parametric one way ANOVA followed by Dunn's post-hoc test were performed, accordingly. Data are presented as box plots. Line in the box, median value; box edges, 25th to 75th centiles; whiskers, range of values;. *p<0.05; ***p<0.001 versus baseline.
